# The Household Resistome: Frequency of β-Lactamases, Class 1 Integrons, and Antibiotic-Resistant Bacteria in the Domestic Environment and Their Reduction during Automated Dishwashing and Laundering

**DOI:** 10.1128/AEM.02062-20

**Published:** 2020-11-10

**Authors:** Laura Schages, Ralf Lucassen, Florian Wichern, Rainer Kalscheuer, Dirk Bockmühl

**Affiliations:** a Rhine-Waal University of Applied Sciences, Faculty of Life Sciences, Kleve, Germany; b Heinrich Heine University Düsseldorf, Institute of Pharmaceutical Biology and Biotechnology, Düsseldorf, Germany; University of Manchester

**Keywords:** antibiotic resistance, household, β-lactamases, class 1 integron, multiresistance, domestic environment

## Abstract

Households provide a habitat for bacteria originating from humans, animals, foods, contaminated clothes, or other sources. Thus, bacteria carrying antibiotic resistance genes (ARGs) may be introduced via household members, animals, or the water supply from external habitats into private households and vice versa. Since data on antibiotic resistance (ABR) in the domestic environment are limited, this study aimed to determine the abundance of β-lactamase, mobile colistin resistance, and class 1 integron genes and the correlation of their presence and to characterize phenotypically resistant strains in 54 private households in Germany. Additionally, the persistence of antibiotic-resistant bacteria during automated dishwashing compared to that during laundering was assessed. Shower drains, washing machines, and dishwashers were sampled and analyzed using quantitative real-time PCR. Resistant strains were isolated, followed by identification and antibiotic susceptibility testing using a Vitek 2 system. The results showed a significantly higher relative ARG abundance of 0.2367 ARG copies/16S rRNA gene copies in shower drains than in dishwashers (0.1329 ARG copies/16S rRNA gene copies) and washing machines (0.0006 ARG copies/16S rRNA gene copies). *bla*_CMY-2_, *bla*_ACT/MIR_, and *bla*_OXA-48_ were the most prevalent ARG, and *intI1* occurred in 96.3% of the households, while no *mcr* genes were detected. Several β-lactamase genes co-occurred, and the resistance of bacterial isolates correlated positively with genotypic resistance, with carbapenemase genes dominating across isolates. Antibiotic-resistant bacteria were significantly reduced during automated dishwashing as well as laundering tests and did not differ from susceptible strains. Overall, the domestic environment may represent a potential reservoir of β-lactamase genes and β-lactam-resistant bacteria, with shower drains being the dominant source of ABR.

**IMPORTANCE** The abundance of antibiotic-resistant bacteria and ARGs is steadily increasing and has been comprehensively analyzed in natural environments, animals, foods, and wastewater treatment plants. In this respect, β-lactams and colistin are of particular interest due to the emergence of multidrug-resistant Gram-negative bacteria. Despite the connection of private households to these environments, only a few studies have focused on the domestic environment so far. Therefore, the present study further investigated the occurrence of ARGs and antibiotic-resistant bacteria in shower drains, washing machines, and dishwashers. The analysis of the domestic environment as a potential reservoir of resistant bacteria is crucial to determine whether households contribute to the spread of ABR or may be a habitat where resistant bacteria from the natural environment, humans, food, or water are selected due to the use of detergents, antimicrobial products, and antibiotics. Furthermore, ABR could limit the options for the treatment of infections arising in the domestic environment.

## Abstract (precis)

The abundance of antibiotic-resistant bacteria and ARGs is steadily increasing and has been comprehensively analyzed in natural environments, animals, foods, and wastewater treatment plants. In this respect, β-lactams and colistin are of particular interest due to the emergence of multidrug-resistant Gram-negative bacteria. Despite the connection of private households to these environments, only a few studies have focused on the domestic environment so far. Therefore, the present study further investigated the occurrence of ARGs and antibiotic-resistant bacteria in shower drains, washing machines, and dishwashers. The analysis of the domestic environment as a potential reservoir of resistant bacteria is crucial to determine whether households contribute to the spread of ABR or may be a habitat where resistant bacteria from the natural environment, humans, food, or water are selected due to the use of detergents, antimicrobial products, and antibiotics. Furthermore, ABR could limit the options for the treatment of infections arising in the domestic environment.

## INTRODUCTION

Infections caused by multidrug-resistant (MDR) Gram-negative bacteria, such as *Enterobacteriaceae* or Pseudomonas aeruginosa, occur worldwide (1–3). In this respect, resistance to β-lactams and colistin is of great concern since these drugs are considered critically important antimicrobials for human medicine (4). Therefore, the increasing number and the diversity of β-lactamase (*bla*) genes (5) and mobile colistin resistance (*mcr*) genes (6) in pathogenic bacteria dramatically limit the options for treatment with the available antibiotics (7, 8). The *bla* and *mcr* genes are often located on mobile genetic elements, such as plasmids, transposons, or integrons (9–12), enabling their horizontal and vertical transfer between bacteria (13). Class 1 integrons (*intI1*) have been identified to be an indicator of pollution with heavy metals, antibiotics, or personal care products and have already been used as a marker for the dissemination of antibiotic resistance genes (ARGs) (14, 15), since they are frequently connected with the carriage of multiple ARGs (11, 16).

Besides acquired antibiotic resistance, intrinsic resistances to antibiotics occur independently of selective pressure or gene transfer and additionally limit the options for the treatment of infections caused by Gram-negative bacteria (17). Common mechanisms of intrinsically resistant bacteria are MDR efflux pumps or reduced uptake of antibiotics, but chromosomally encoded ARGs are involved as well (18, 19). Stenotrophomonas maltophilia is especially an opportunistic nosocomial pathogen and possesses intrinsic multidrug resistances to a large array of antibiotics (20, 21).

Antibiotic-resistant bacteria and ARGs have been comprehensively analyzed in humans, animals, food, soil, water, and wastewater (22–28). However, it seems reasonable that bacteria carrying ARGs may be introduced into private households by contaminated clothes, skin, foodstuffs, or other sources, and their selection may be promoted by antibiotics and antibacterial agents used in cleaning agents or personal care products (29–31). Since domestic wastewater must be considered an important component of wastewater, households could also play a role in the dissemination of antibiotic-resistant bacteria and ARGs. However, data on the domestic environment are limited (15, 32, 33), although antibiotic-resistant bacteria in domestic appliances, such as washing machines or dishwashers, may pose a potential health risk to inhabitants. Cross-contaminations may occur (34–37), since infections in general can originate from domestic surfaces (38), and Schmithausen et al. (37) found evidence for the transfer of an extended-spectrum β-lactamase (ESBL)-producing Klebsiella oxytoca strain from a washing machine via textiles to newborns. Studies have also shown that biofilms are prevalent in washing machines and dishwashers (39, 40) and that their detachment may result in contaminations on the cleaned items. Furthermore, cross-contaminations may be caused by the detachment of bacterial cells from dirty laundry/kitchen utensils during the cleaning process and their reattachment to the washed clothes/dishes (36, 41). Moreover, antibiotic-resistant bacteria may be more persistent during automated dishwashing or laundering, since the stress response and heat intolerance have been associated with antibiotic resistance (ABR) (42, 43).

In an earlier study, the occurrence of *bla* genes in domestic appliances was analyzed, and it was found that *bla* genes occurred in 79% of the washing machines and in 96% of the dishwashers, with *ampC*- and OXA-type genes dominating (32). However, phenotypic resistance was not determined, and the *bla* genes were analyzed only qualitatively. Although the impact of domestic laundering was assessed in this study, we focused on different conditions and used a newly developed method to be able to analyze the rinsing cycle as well (44). Furthermore, we investigated the effect of automated dishwashing on antibiotic-resistant bacteria. On the basis of these previous observations, the present study aimed to enhance our understanding of ABR in households by quantifying the abundance of *bla*, *mcr*, and *intI1* genes in washing machines, dishwashers, and shower drains. We hypothesized (i) that ARGs and antibiotic-resistant bacteria are highly abundant in households, with the highest diversity being found in shower drains; (ii) that *bla* genes frequently co-occur in household samples; and (iii) that similar reductions of antibiotic-resistant bacteria are achieved during automated dishwashing and laundering.

## RESULTS

### Frequency distribution and co-occurrence of *bla* and *intI1* genes in households.

**FIG 1:**
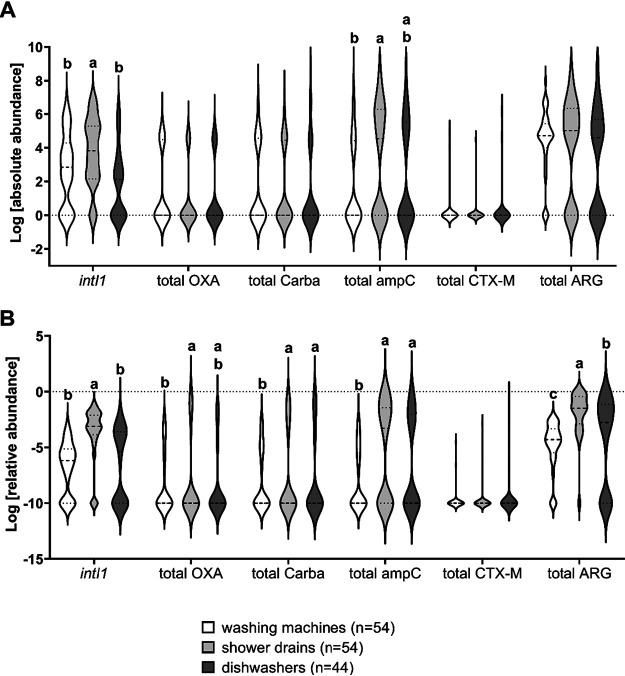
Violin plots showing the distribution and variation of the absolute (A) and relative (B) abundances of *intI1* and *bla* genes in washing machine (WM), shower drain (SD), and dishwasher (DW) samples. The total for each gene group is presented: OXA genes (*bla*_OXA-58_ and *bla*_OXA-23_), carbapenemase genes (Carba; *bla*_OXA-48_, *bla*_GES_, *bla*_KPC_, and *bla*_VIM_), AmpC genes (*bla*_CMY-2_, *bla*_FOX_, *bla*_ACT/MIR_, and *bla*_DHA_), and CTX-M genes (*bla*_CTX-M-1_ and *bla*_CTX-M-9_). In panel B, the values for samples without an ARG (relative abundance = 0) are presented as −10. Different letters indicate significant differences at a *P* value of *≤*0.05 between SD, WM, and DW samples in each group. Where no letters are shown, no significant differences were detected.

In total, 207 β-lactamase (*bla*) genes were identified in shower drains (SD; *n* = 54), washing machines (WM; *n* = 54), and dishwashers (DW; *n* = 44), and 12 of the 13 targeted β-lactamase types were detected, while no mobile colistin resistance genes (*mcr-1* and *mcr-2*) occurred. *bla*_VIM_ and *bla*_DHA_ were identified in only two SD samples, and *bla*_CTX-M-9_ was detected once in a WM, whereas the metallo-β-lactamase NDM occurred in none of the samples. The most frequently detected ARG was *bla*_CMY-2_, followed by *bla*_ACT/MIR_ and *bla*_OXA-48_. The absolute abundance of the *intI1* and AmpC β-lactamase genes was significantly higher (*P = *0.0385 and *P = *0.0008, respectively) in SD samples (mean log values, 3.77 ± 1.99 for *intI1* and 3.37 ± 3.16 for *ampC*) than in WM samples (mean log values, 2.71 ± 2.14 for *intI1* and 1.79 ± 2.55 for *ampC*), and the absolute abundance was significantly higher in SD and WM samples than in DW samples only for AmpC β-lactamase genes (*P = *0.0001) (mean log value, 2.50 ± 3.00). In contrast, the relative abundance of AmpC β-lactamase genes (*P < *0.0001 for SD samples and *P = *0.0226 for DW samples), carbapenemase genes (*P*= 0.0015 for SD samples and *P = *0.0022 for DW samples), and total ARGs (*P < *0.0001 for SD samples and *P = *0.0004 for DW samples) was significantly higher in SD samples (mean log values, −3,69 ± 2.48 for *intI1*, −5.29 ± 4.34 for *ampC*, −7.58 ± 3.82 for carbapenemase genes, and −1.91 ± 2.07 for total ARGs) and DW samples than in WM samples, and the relative abundance of *intI1* (*P < *0.0001) and total ARGs (*P = *0.0002) was significantly higher in SD samples than in DW samples, while the abundance of carbapenemase genes (mean log value, −7.79 ± 3.74) was slightly higher in DW samples than in the other samples (Fig. 1).

The number of ARGs detected in individual samples ranged from zero to five, and the SD samples revealed the highest diversity, with 24.1% containing three or more *bla* genes, while in 40.9% of DW samples and 33.3% of WM samples, no *bla* gene was detected (Table 1). Most ARGs were detected in SD (39.1%), followed by WM (33.3%) and DW (27.5%). An absolute abundance of ARGs of 1.53 × 10^7^ gene copies ml^−1^ in SD samples, 2.52 × 10^6^ gene copies ml^−1^ in WM samples, and 2.74 × 10^7^ gene copies ml^−1^ in DW samples was determined. In contrast, the relative abundance was the highest in SD samples (0.2367 ARG copies/16S rRNA gene copies), followed by DW samples (0.1329 ARG copies/16S rRNA gene copies) and WM samples (0.0006 ARG copies/16S rRNA gene copies).

**TABLE 1:** Co-occurrence of *bla* genes in shower drain, washing machine, and dishwasher samples

No. of *bla* genes	% of samples		
	Shower drains (*n* = 54)	Washing machines (*n* = 54)	Dishwashers (*n* = 44)
0	29.6	33.3	40.9
1	24.1	31.5	20.5
2	22.2	22.2	20.5
≥3	24.1	12.9	18.2

**FIG 2:**
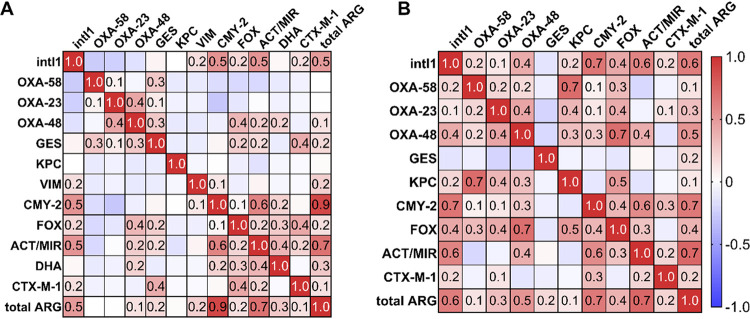
Correlation matrix showing the co-occurrence of *bla* genes in shower drains (A) and dishwashers (B). Red indicates a strong positive correlation, while blue indicates a strong negative correlation, and a correlation with an *r* value of ≥0.3 is statistically significant. *bla* genes in washing machine samples did not correlate significantly.

The co-occurrence of *bla* genes was explored using the Spearman correlation, and the absolute abundance of total ARGs, *bla*_CMY-2_, and *bla*_ACT/MIR_ correlated strongly with that of *intI1* in SD samples (*P = *0.0002, *P < *0.0001, and *P < *0.0001, respectively) and DW samples (*P < *0.0001). Furthermore, the absolute abundance of *bla*_CMY-2_ and *bla*_ACT/MIR_ correlated strongly in SD samples (*P < *0.0001) and DW samples (*P < *0.0001), while, inter alia, absolute abundance of *bla*_OXA-23_ and *bla*_OXA-48_ (*P = *0.0011 for SD samples, *P = *0.0030 for DW samples) as well as *bla*_OXA-48_ and *bla*_FOX_ (*P = *0.0094 for SD samples, *P < *0.0001 for DW samples) revealed a positive correlation (Fig. 2). In DW samples, absolute abundance of *bla*_OXA-58_ and *bla*_KPC_ (*P < *0.0001) correlated strongly, whereas a moderate positive correlation of absolute abundance of *bla*_GES_ and *bla*_CTX-M-1_ (*P = *0.0027) as well as *bla*_ACT/MIR_ and *bla*_DHA_ (*P = *0.0076) was observed in SD samples. Furthermore, ARGs co-occurred in bacterial isolates as well, resulting in more than one *bla* gene in 19 of 41 isolates. Carbapenemase and CTX-M genes, carbapenemase and *ampC* genes, as well as CTX-M and *ampC* genes were detected within the same strain (see Tables S1 and S2 in the supplemental material). Among others, an MDR Escherichia coli strain harboring *bla*_OXA-48_ and *bla*_CTX-M-1_ in an SD sample, a Pseudomonas putida strain with *bla*_OXA-48_, *bla*_GES_, and *bla*_CTX-M-1_ in a WM sample, and an ESBL-producing Enterobacter cloacae strain harboring *bla*_CTX-M-1_ and *bla*_ACT/MIR_ in a DW sample were isolated.

### Abundance of antibiotic-resistant bacteria in household samples.

A total of 273 strains was isolated from household samples using low concentrations of antibiotics (Table 2). Stenotrophomonas maltophilia was predominant among the isolates from SD and DW samples, while Pseudomonas aeruginosa slightly dominated in WM samples. However, no significant differences were determined (*P ≤ *0.05). In contrast, species of the family *Enterobacteriaceae* were the second most abundant in SD and DW samples. Except for the abundance of *Enterobacteriaceae* and *Pseudomonas* spp., bacterial species were distributed equally among SD, WM, and DW samples. However, fewer strains were isolated from DW samples than from the other samples, due to no detectable growth in supplemented tryptic soy broth (TSB).

**TABLE 2:** Percentage of different bacterial species isolated from broth cultures inoculated with imipenem, cefotaxime, or colistin after suspending swab samples from shower drains, washing machines, and dishwashers in tryptic soy broth

Bacterial species	% of samples		
	Shower drains (*n* = 129)	Washing machines (*n* = 83)	Dishwashers (*n* = 61)
*Enterobacteriaceae*	21.7	10.8	21.3
Pseudomonas aeruginosa	9.3	19.3	3.3
Other *Pseudomonas* spp.	12.4	15.7	11.5
Stenotrophomonas maltophilia	27.9	18.1	37.7
Burkholderia cepacia	7.0	8.4	6.6
Acinetobacter spp.	1.6	3.6	1.6
*Aeromonas* spp.	3.9	1.2	3.3
Others^*a*^	10.9	18.1	8.2
Not identified	5.4	4.8	6.6

a Others include Achromobacter denitrificans, Achromobacter xylosoxidans, Brevundimonas diminuta, Chryseobacterium indologenes, Delftia acidovorans, Ochrobactrum anthropi, Roseomonas gilardii, Shewanella putrefaciens, and Sphingomonas paucimobilis.

**FIG 3:**
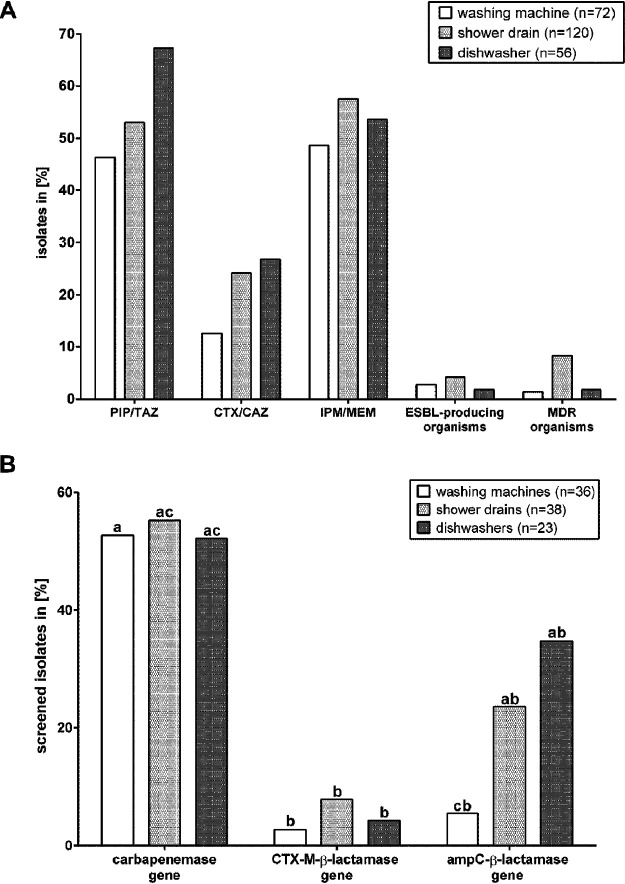
Percentage of β-lactam-resistant bacterial species (A) and *bla* genes detected in screened isolates (B) from the shower drains (SD), washing machines (WM), and dishwashers (DW) of 54 different households. Resistances and the ESBL phenotype were determined using a Vitek 2 system (bioMérieux), and MDR bacteria were classified according to international recommendations. Different letters indicate significant differences at a *P* value of *≤*0.05 between SD, WM, and DW (A) and carbapenemase, CTX-M, and *ampC* genes (B). Where no letters are shown, no significant differences were detected.

The determination of antibiotic resistances by use of the Vitek 2 system failed for 11 isolates from WM samples, 9 isolates from SD samples, and 5 isolates from DW samples due to no detectable growth. The rate of insusceptibility to piperacillin-tazobactam (PIP/TAZ) was the highest in DW samples (67.3%), while insusceptibility to imipenem/meropenem (IPM/MEM) dominated across SD and WM samples (Fig. 3A). Resistance to cefotaxime/ceftazidime (CTX/CAZ) was higher in SD and DW samples than in WM samples. MDR strains were classified according to international recommendations (45). Most of the identified species were environmental opportunistic pathogens, and although MDR and ESBL-producing bacteria were isolated from all samples without significant differences between SD, WM, and DW samples (*P ≤ *0.05), their contribution to resistant bacteria was the highest in the SD samples (8.3%).

Quantitative real-time PCR (qPCR) for the detection of *bla* and *mcr* genes was performed with the DNA extracted from the isolates, and in the case of 33 of the 99 isolates, the resistance profile of the phenotype was in accordance with the ARGs detected. Most *bla* genes were identified in strains of the *Enterobacteriaceae*, followed by strains of the *Pseudomonadaceae*. Carbapenemase genes were predominantly detected, while no ARGs were detected in approximately 50% of the isolates. Except for *ampC* genes, SD samples revealed the highest percentage of strains carrying ARGs (Fig. 3B). Of the strains which were screened for *bla* and *mcr* genes, all MDR bacteria carrying ARGs were isolated from SD (Table S1 and S2). Furthermore, the percentage of carbapenemase genes was significantly higher (for SD samples, *P = *0.0340; for WM samples, *P = *0.0285; for DW samples, *P = *0.0285) than that of CTX-M-β-lactamase genes in all samples (mean difference, 48.4% ± 1.4%).

**FIG 4:**
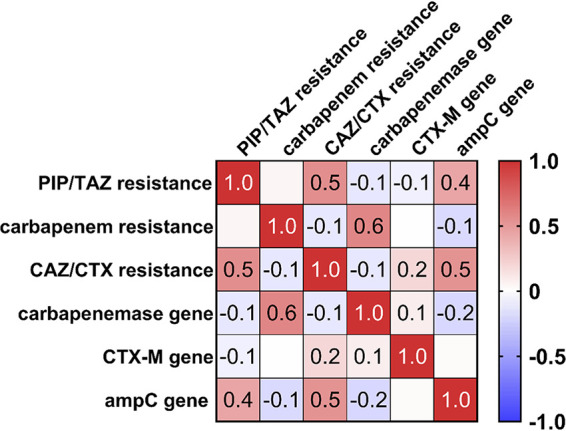
Spearman correlation (*P ≤ *0.05) of genotype (carbapenemase genes *bla*_OXA-58_, *bla*_OXA-23_, *bla*_OXA-48_, *bla*_GES_, *bla*_KPC_, and *bla*_VIM_; *ampC* genes *bla*_CMY-2_, *bla*_FOX_, *bla*_ACT/MIR_, and *bla*_DHA_; and CTX-M genes *bla*_CTX-M-1_ and *bla*_CTX-M-9_) with phenotypic resistance (to piperacillin-tazobactam [PIP/TAZ], carbapenems, and ceftazidime [CAZ] and/or cefotaxime [CTX]) in screened isolates from households.

The Spearman correlation of the carbapenemase, CTX-M, and *ampC* genes with phenotypic resistance against carbapenems, ceftazidime (CAZ) and/or cefotaxime (CTX), and piperacillin-tazobactam (PIP/TAZ) was performed to identify the relevance of the ARGs detected (Fig. 4). The high percentage of carbapenem-resistant strains was confirmed by the detection of carbapenemase genes in more than 50% of the isolates and the strong positive correlation (*P < *0.0001) of carbapenemase genes and carbapenem resistance. PIP/TAZ and CAZ/CTX resistance correlated strongly with the presence of *ampC* genes (*P < *0.0001). Even though the occurrence of CTX-M genes correlated positively with CAZ/CTX resistance as well, the correlation determined was weak (*P = *0.0631).

### Effect of laundering and automated dishwashing on antibiotic-resistant strains.

To evaluate the persistence of antibiotic-resistant bacteria in response to laundry procedures compared to that of susceptible strains, artificially contaminated cotton swatches were washed in a lab-scale washing machine (Rotawash), simulating household-relevant laundering parameters. An activated oxygen bleach (AOB)-free detergent for the main wash and benzalkonium chloride (BAC) for the rinsing cycle were chosen, since the use of liquid or color detergents (without AOB) has increased steadily in private households (46) and BAC is commonly used in rinse aids. Textile swatches were artificially contaminated, resulting in initial microbial counts (expressed as the number of CFU milliliter^−1^ of recovery fluid) of 2.72 × 10^7^ CFU ml^−1^ for E. coli, 3.29 × 10^7^ CFU ml^−1^ for carbapenemase-producing E. coli, 2.25 × 10^7^ CFU ml^−1^ for Klebsiella pneumoniae, 3.01 × 10^7^ CFU ml^−1^ for ESBL-producing K. pneumoniae, 7.45 × 10^7^ CFU ml^−1^ for Staphylococcus aureus, and 3.38 × 10^7^ CFU ml^−1^ for methicillin-resistant Staphylococcus aureus (MRSA). Although both susceptible and resistant strains were tested, no significant differences were revealed, except in tests with BAC between the susceptible and antibiotic-resistant strains of the same species. Therefore, only the results for the antibiotic-resistant strains are shown, and the results for the susceptible strains are provided in the supplemental material (Fig. S1).

**FIG 5:**
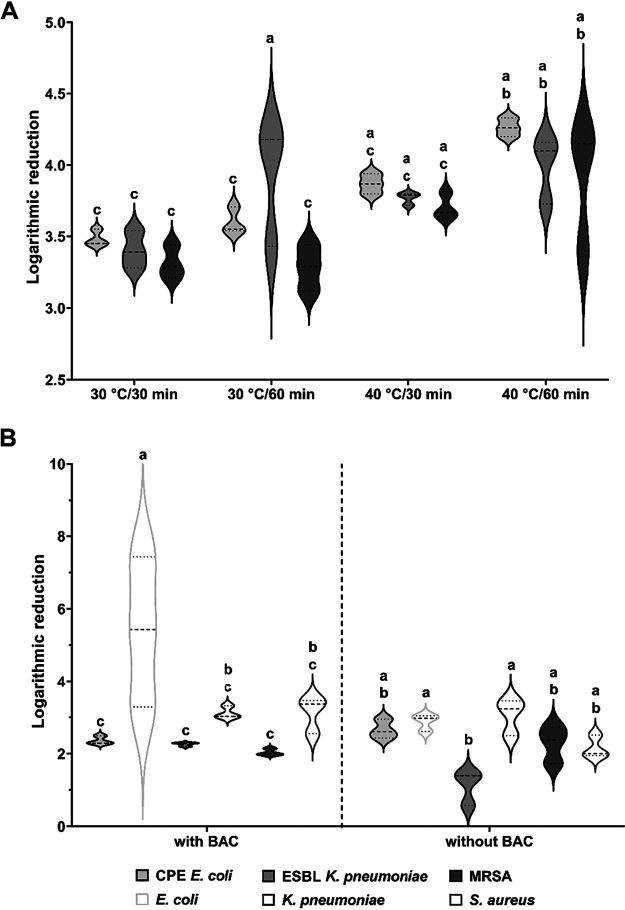
Impact of a main wash with AOB-free detergent on antibiotic-resistant strains (A) and a rinsing cycle with and without benzalkonium chloride (BAC) (B) in the laundering process, simulated using a lab-scale washing machine (Rotawash), on resistant and susceptible strains of E. coli, K. pneumoniae, and S. aureus. Violin plots show the distribution and variation, and different letters indicate significant differences at a *P* value of *≤*0.05. CPE, carbapenemase-producing *Enterobacteriaceae*.

Tests performed with AOB-free detergent in the lab-scale washing machine (Fig. 5A) revealed only minor differences between the logarithmic reduction (LR) of carbapenemase-producing E. coli (3.80 ± 0.35), ESBL-producing K. pneumoniae (3.78 ± 0.27), and MRSA (3.55 ± 0.31). In general, the results indicated an increasing efficacy of laundering with increasing time and temperature, revealing significantly higher reductions of all strains by laundering at 40°C for 60 min compared to that achieved in tests at 30°C (mean *P* value = 0.00598). The tests of the rinsing cycle showed that the effect of 0.02% BAC was negligible, except for the significantly higher reduction (*P = *0.0002) of the susceptible E. coli strain (Fig. 5B). However, the highest reductions achieved were in the range of 45% to 57% at 40°C and a main wash of 60 min.

**FIG 6:**
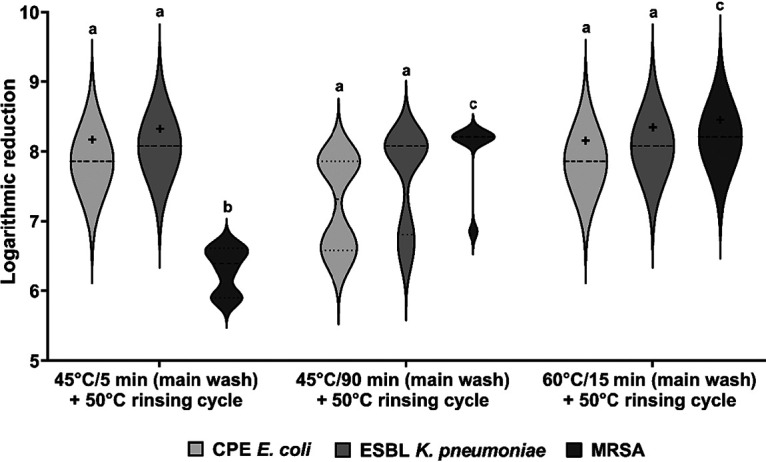
Impact of automated dishwashing on antibiotic-resistant strains of E. coli, K. pneumoniae, and S. aureus with detergent. The different values for the maximum LR (+, indicating a complete reduction of the microbial load in the case of 45°C for 5 or 90 min and 60°C for 15 min) were obtained due to different initial loads on the biomonitors. The violin plots show the distribution and variation, and different letters indicate significant differences at a *P* value of *≤*0.05.

The same test strains were used to determine the effect of automated dishwashing on antibiotic-resistant strains. The initial microbial counts of the contaminated biomonitors were 6.82 × 10^7^ CFU ml^−1^ for E. coli, 7.18 × 10^7^ CFU ml^−1^ for carbapenemase-producing E. coli, 1.40 × 10^7^ CFU ml^−1^ for K. pneumoniae, 1.20 × 10^8^ CFU ml^−1^ for ESBL-producing K. pneumoniae, 1.55 × 10^8^ CFU ml^−1^ for S. aureus, and 1.62 × 10^8^ CFU ml^−1^ for MRSA. In order to simulate household-relevant conditions, the following programs were tested using the reference detergent: a 5-min main wash at 45°C (short program), a 15-min main wash at 60°C (standard program), and a 90-min main wash at 45°C (ecoprogram). Again, only the results for the antibiotic-resistant strains are shown since the results between antibiotic-resistant strains (Fig. 6) and antibiotic-susceptible strains (Fig. S2) did not differ significantly.

Regarding all antibiotic-resistant strains, a complete reduction was achieved at 60°C, and only the logarithmic reduction of MRSA was significantly lower (*P < *0.0001) at 45°C for 5 min (Fig. 6). Even under the conditions of the short program, the maximum LR (exceeding the detection limit) was reached for all strains except MRSA (6.3 ± 0.33; maximum LR, 8.2), while the ecoprogram revealed lower logarithmic reductions, but with all programs, the reductions were at least above 75%.

## DISCUSSION

Previous studies revealed the presence of *bla* genes (32) and the correlation of multiresistance with *intI1* (15) in the domestic environment, but no studies on the quantitative prevalence of *bla* genes and their co-occurrence in private households, especially in relation to phenotypic resistance, have been conducted so far. SD, WM, and DW provide good habitats for microbial communities with a steady nutrient supply in a humid environment, promoting bacterial growth and biofilm formation (39, 47–49).

### Prevalence and co-occurrence of ARGs and antibiotic-resistant bacteria in household samples.

The results obtained showed a strong deviation in the number of all *bla* genes across SD, WM, and DW samples due to the very high absolute abundances of ARGs (e.g., 4.04 × 10^8^ copies ml^−1^ of carbapenemase genes in a DW sample), although specific ARGs were not detected in a large number of samples (e.g., 95.5% of all household samples, in the case of CTX-M genes). However, the relative abundance of ARGs, except for CTX-M genes, was higher in SD samples than in the other samples, indicating a higher frequency of ARGs in SD. AmpC β-lactamase genes occurred predominantly in all samples and dominated in a previous study of WM and DW as well (32).

Correlation analysis revealed a significantly positive co-occurrence of *bla* genes, such as *bla*_CMY-2_ and *bla*_ACT/MIR_, *bla*_OXA-23_ and *bla*_OXA-48_, or *bla*_GES_ and *bla*_CTX-M-1_, in SD and DW. The targeted *bla* genes in this study are usually plasmid encoded or part of class 1 integrons on transferable plasmids. The OXA-23-, OXA-58-, and OXA-48-like β-lactamases (50), CTX-M-1 and members of the CTX-M-9 family, and the *ampC* genes *bla*_CMY-2_, *bla*_FOX_, *bla*_ACT_, *bla*_MIR_, and *bla*_DHA_ are all mainly plasmid borne (9, 51). As part of transferable plasmids, the carbapenemases *bla*_GES_ and *bla*_KPC_ and the metallo-β-lactamases *bla*_VIM_ and *bla*_NDM_ have been associated with *Enterobacteriaceae* and *Pseudomonadaceae* (1). Thus, the correlation of the ARGs could be connected to their location on mobile genetic elements or to the different bacterial strains within samples. Furthermore, *bla*_VIM_, *bla*_GES_, *bla*_CTX-M-1_, and *bla*_CTX-M-9_ have been identified within class 1 integrons (51). However, these genes correlated only weakly with *intI1* in SD and DW samples, in contrast to *bla*_CMY-2_, *bla*_ACT/MIR_, or *bla*_OXA-48_. Although these ARGs have not been detected within class 1 integrons so far, the *intI1* and *bla* genes could be located on the same plasmid or on different plasmids harbored by the same strain. Furthermore, class 1 integrons frequently occur in diverse pathogenic and commensal isolates of *Enterobacteriaceae* and other Gram-negative bacteria, and thus, their presence may correlate with that of the most abundant *bla* genes due to their high prevalence in the species detected. In the majority of SD, WM, and DW samples and in 43.9% of isolates carrying *bla* genes, more than one *bla* gene was detected. This is supported by the findings of Ju et al. (52), who revealed the co-occurrence of ARGs that shared the same resistance mechanism, since the selective pressure of the same antibiotic resulted in the coselection of the same ARG types. Studies revealed that gene cassettes within class 1 integrons carry carbapenemase, ESBL, and AmpC and OXA β-lactamase genes (53–55), and the abundance of total ARGs and *intI1* correlated strongly in our study. However, since we have no proof of the location of the *bla* genes, it is unknown whether or not the genes are located on class 1 integrons. The isolation of bacteria harboring both carbapenemase and CTX-M genes is worrisome, since options for the treatment of infections caused by strains conferring resistances to both cephalosporins and carbapenems may be drastically limited.

The bacterial community in SD, WM, and DW usually comprises environmental bacteria, including, among others, *Enterobacteriaceae* and *Pseudomonadaceae* (32, 39, 48), which, apart from Stenotrophomonas maltophilia, were the predominant species in this study as well. The species identified are known to harbor *bla* genes (1, 7, 56), and the isolation of clinically relevant species, such as MDR P. aeruginosa and ESBL-producing E. coli strains harboring carbapenemase genes, suggests that the domestic environment may be a potential reservoir for the spread of β-lactam resistance. The positive correlation of carbapenemase and AmpC β-lactamase genes with carbapenem and cefotaxime/ceftazidime resistance, respectively, shows that the phenotypic resistance is most likely based on β-lactamase production. Interestingly, in 21 out of 54 households, at least one *bla* gene detected was the same in all samples (12 households), in SD and DW samples (5 households), in SD and WM samples (4 households), or in WM and DW samples (2 households). The connection of the sampling points to the water supply system (57) and the growth of biofilms harboring diverse microbial communities on inner pipe surfaces (39, 58) may contribute to the spread of ARGs and antibiotic-resistant bacteria from/to and within households. In addition to the possible inlet of antibiotic-resistant bacteria via the water supply system, a transfer within the same household by household members or contaminated eating utensils/laundry is likely as well (59–61) and has been shown by Schmithausen et al. (37) in a WM. However, compared to wastewater treatment plants, where domestic, industrial, and clinical sewage accumulates, the abundance of ARG and MDR bacteria in the domestic environment is much lower (28).

### Shower drains as an important source of ARGs and ABR in private households.

The significantly higher relative abundance (*intI1* and the total of carbapenemase, OXA β-lactamase, and AmpC β-lactamase genes) in SD samples than in WM samples and the partially significantly higher relative abundance in SD samples than in DW samples may indicate a higher resistance potential. Shower drains are known to be prone to the formation of biofilms, which are more resistant to environmental factors (such as exposure to antibiotics) and promote horizontal gene transfer and, thus, ABR (62–64). Although biofilms can also form in WM and DW (39), the higher temperatures and the drying of surfaces after use enable only well-adapted species to grow, while SD supply less extreme temperatures and a constant humid environment. For instance, it has been shown that Gram-positive bacteria dominate in DW (40) and that in WM biofilms occur more frequently in the inner parts of the device (39), which may explain the lower ARG abundance in DW and WM samples than in samples from SD. Therefore, the high abundance of ARGs, especially of *ampC* genes (58), may be related to biofilms in SD. Besides biofilm formation, the higher ARG level in SD could also be connected to the human body as a carrier of ABR. Antibiotic-resistant bacteria have been detected on the skin (65), in the gut (66, 67), and in the oral cavity (68), and thus, the detachment of these bacteria during showering seems likely. Domestic drains are colonized with *Pseudomonadaceae* and coliform bacteria (48), which dominated in the SD samples investigated and which are associated with β-lactamases, multidrug resistance, and nosocomial outbreaks originating from contaminated sinks (69, 70). We found that strains harboring *bla* genes and ESBL-producing and MDR bacteria dominated in SD, which indicates a higher potential for resistance in these strains than in strains from WM and DW as well. This is supported by the identification of shower and sink drains as hot spots for *intI1* compared to the likelihood of its identification in other household areas by Lucassen et al. (15) and by a study of the frequency of antibiotic-resistant bacteria in households of Marshall et al. (33), who revealed the highest titers of antibiotic-resistant bacteria in sink drains. Thus, the dissemination of antibiotic-resistant bacteria colonizing the human body to SD and their selective accumulation due to the exposure to the biocides used in cleaning agents and personal care products (33, 71) could explain their higher frequency in SD than in WM and DW.

### Effect of automated dishwashing and laundering on antibiotic-resistant bacteria.

The analysis of the impact of laundering and automated dishwashing on antibiotic-resistant bacteria showed that their efficacy increased with increasing duration and temperature. The rates of reduction of resistant strains and nonresistant strains in both WM and DW were quite similar, and only MRSA and S. aureus revealed a higher persistence than other species, which was determined for both resistant and nonresistant strains in other studies as well (32, 72). However, the abundance of all antibiotic-resistant test strains was reduced significantly, and since bacteria on textiles usually originate from the human microbiome or the environment, the human health risk should be rather low. To achieve higher reductions during laundering when a sufficient level of hygiene must be guaranteed, higher temperatures or the use of a heavy-duty detergent containing a bleaching agent may be necessary (46), since bacterial counts of approximately 10^3^ CFU ml^−1^ were detected even after laundering at 40°C for 60 min. Furthermore, laundering at lower temperatures when using bleach-free detergents may lead to cross-contaminations of the laundry or the WM (35, 73) with antibiotic-resistant bacteria, possibly enabling their transfer within the household or via the gray water in other environments. Thus, for critical cases, such as risk groups being cared for at home or individuals with acute infections, the use of higher temperatures and AOB should be preferred (34). Besides, no significantly higher reduction of antibiotic-resistant bacteria was observed when using 0.02% BAC in the rinsing cycle. However, further analyses of the rinsing cycle are necessary to determine the effect of BAC, since the conditions tested in the present study were limited. No bacterial growth was detected in dishwashers after test runs, except with the ecoprogram; thus, cleaned dishes do not seem to be a source of the tested strains. However, antibiotic-resistant bacteria were isolated from rubber door seals and DW sieves and have already been identified to be a source of pathogenic and opportunistic pathogenic bacteria and fungi (40, 49, 74). Zupančič et al. (49) determined a low phenotypic ABR potential for strains from DW, a finding which was confirmed by our study, since fewer resistant strains were isolated from DW than from SD and WM. Nevertheless, DW samples revealed a high absolute abundance of ARGs, and the strains that were isolated still harbored *bla* genes and thus can be considered a potential source of antibiotic-resistant bacteria as well.

Despite the prevalence of ARGs and the partial survival of antibiotic-resistant bacteria during automated dishwashing and laundering, the actual risk posed by dishes and laundry items is not known. Studies showed that drying and ironing of laundry additionally contribute to the reduction of bacteria on textiles and even result in their complete removal (75–77). This effect should apply for antibiotic-resistant bacteria as well, since no significant differences between susceptible and resistant strains during laundering were obtained. The dishwasher sump and rubber seal provide good conditions for microbial growth, while the walls were revealed to have only low levels of contaminations (40, 49, 78). Therefore, it can be assumed that dishes cleaned in the dishwasher show similarly low bacterial loads. These studies indicate that the actual risk of infection with both resistant and nonresistant bacteria should be considerably low.

### Conclusion.

The results obtained in this study substantiate that the domestic environment represents a potential reservoir of *bla* genes and β-lactam-resistant bacteria. We found that ARGs co-occurred in household samples, indicating that bacterial species harboring multiple *bla* genes or bacterial communities harboring multiple β-lactam-resistant species are frequent in SD, WM, and DW. This evidence was supported by the detection of various *bla* genes in the bacterial isolates. Moreover, our data show that SD revealed a higher abundance of bacteria with β-lactam resistance than WM and DW in households, with a higher frequency of bacteria harboring *bla* genes and MDR and ESBL-producing bacteria. Although laundering and automated dishwashing significantly reduced the amounts of antibiotic-resistant bacteria, low bacterial counts were still detected, especially after laundering. Thus, a transfer of antibiotic-resistant bacteria via contaminated laundry or the gray water of WM in other environments may be possible. However, further studies of the domestic environment are needed to confirm the possible risk of dissemination of ARGs and antibiotic-resistant bacteria and to determine whether households contribute to the spread of ABR or are a habitat where resistant bacteria from the environment, humans, food, or water accumulate.

## MATERIALS AND METHODS

### Sampling and sample preparation.

Samples of shower drains, dishwasher sumps and sieves, detergent trays, and rubber door seals of washing machines were taken from 54 households in North Rhine-Westphalia, Germany, between September 2018 and June 2019. The households were chosen to get a broad spectrum of different conditions in order to reflect realistic domestic situations and on the basis of location (focusing on the lower Rhine region). Table 3 provides the main conditions of the participating households. All samples were taken from households without health care or support provided by a health care professional at home. Samples were collected in triplicate by probing the surfaces of the inner tubing of shower drains (SD), the sumps and sieves of dishwashers (DW), or the detergent trays and rubber door seals of washing machines (WM) by the swab method. Swab samples were collected by swabbing the selected surface with several firm vertical and horizontal strokes, using a sterile cotton swab. In order to increase the amount of material extracted and to ensure that the entire surface of the swab was used, the sterile cotton swab was moistened in sterile 0.9% sodium chloride before sampling and was rotated during sampling (79).

**TABLE 3:** Overview of conditions across the households analyzed in the study^*a*^

Condition	No. of households
Single-family house	21
Apartment	33
Households with 1–2 members	29
Households with >2 members	25
Households with members between 20 and 40 yr old	32
Households with members between 40 and 60 yr old	13
Households with members between 60 and 90 yr old	9
Households with children	9

a The age of the children was not included in the categories of the age groups.

Samples were immediately stored at 4°C and prepared within 24 h. Samples collected by the swab method were suspended in 1,000 μl of sterile 0.9% sodium chloride (three swabs were taken and pooled). After centrifugation at 4,800 × *g* and 8°C for 15 min, the supernatant was discarded and the resulting pellet was resuspended in 500 μl of sterile 0.9% sodium chloride (15). The suspended samples were used for further analyses.

### DNA extraction.

For purification of total DNA, a Fast DNA spin kit for soil (MP Bio, Santa Ana, CA, USA) was used according to the manufacturer’s instructions with the following adjustments: instead of 500 mg solid material, 250 μl of suspended sample was applied to the lysing matrix tube. Samples were homogenized twice in a FastPrep-24 instrument for 60 s at 6.0 m/s. All samples were washed twice using the SEWS-M solution provided with the kit. After DNA extraction, quantitative real-time PCR (qPCR) was performed for the detection of resistance genes.

### Detection of *bla*, *mcr*, and *intI1* genes.

To detect *bla* and *mcr* genes, a multiplex qPCR was performed as described by Rehberg et al. (32). The sequences of all primers and probes, the fluorophores, and the quencher used (custom synthesized by Biolegio, Nijmegen, Netherlands) are shown in Table 4. The *bla*_ACT_ and *bla*_MIR_ genes are referred to as *bla*_ACT/MIR_, since the oligonucleotides targeted both. The prepared DNA was amplified using a HotStarTaq master mix kit (Qiagen, Hilden, Germany) and a mix of oligonucleotides and DNA probes labeled with different dyes. Since we used a multiplex assay, the following genes were detected within one reaction: *bla*_OXA-23_ and *bla*_OXA-58_; *bla*_GES_, *bla*_OXA-48_, and *bla*_KPC_; *bla*_CMY-2_ and *bla*_FOX_; *bla*_ACT/MIR_ and *bla*_DHA_; and, in the last mix, *bla*_CTX-M-1_ and *bla*_CTX-M-9_. Different fluorophores were used to enable the differentiation of the *bla* genes, and color compensation objects were created as described in the LightCycler 480 operator’s manual to minimize cross talk between the different channels. Each PCR mix contained 10 μl of 2× HotStarTaq master mix, 7 μl of RNase-free water, 1 μl of oligonucleotide mix (4 μM oligonucleotide, 2 μM probe, 50 μM MgCl_2_), and 2 μl of the DNA template, comprising a final volume of 20 μl. The PCR was performed on a LightCycler 480 instrument (Roche Life Sciences, Mannheim, Germany) using the following conditions: 95°C for 15 min; 45 cycles of denaturation (95°C, 10 s), annealing (60°C, 20 s), and elongation (72°C, 10 s); and a final cycle at 30°C for 30 s. A sample was considered positive if it reached the threshold before cycle 40 or if the number of copies per microliter determined by use of a standard curve was greater than 5 copies/μl. For determination of the 16S rRNA and *intI1* genes, qPCR was performed as described by Lucassen et al. (15). The primers are shown in Table 5.

**TABLE 4:** Primers used for detection of genes encoding β-lactamases (*bla*) and mobile colistin resistance (*mcr*)

Primer or probe	Sequence (5′–3′)^*a*^
KPC-1	GCCGTGCAATACAGTGATAAC
KPC-2	GAACGTGGTATCGCCGATAG
KPC-probe	TexRed-CCGCCGCCAATTTGTTGCTGAAGG-BHQ2
OXA48-1	GTTGGAATGCTCACTTTACTGAA
OXA48-2	TTCGCCCGTTTAAGATTATTGG
OXA48-probe	FAM-ATTCTCATTCCAGAGCACAACTACGCC-BHQ1
GES-1	CTCTGTGAGTCGGCTAGACC
GES-2	CGATCAGCCACCTCTCAATG
GES-probe	HEX-ACACCTGGCGACCTCAGAGATACAACT-BHQ1
VIM-1	GTTTGGTYGCATATCGCAAC
VIM-2	CTTYTCAATCTCCGCGAGAAG
VIM-probe1	FAM-AGCAACTCATCRCCATCACGGACAATG-BHQ1
VIM-probe2	FAM-AACTCGGTGACACGGTGTACTCGTCT-BHQ1
NDM-1	CGACTTATGCCAATGCGTTG
NDM-2	CGGGGTAAAATACCTTGAGC
NDM-probe	HEX-AGCCTGACTTTCGCCGCCAATG-BHQ1
OXA23-1	CCTGATCGGATTGGAGAACC
OXA23-2	GTTCCTGATAGACTGGGACTG
OXA23-probe	FAM-TGGCTTCTCCTAGTGTCATGTCTT-BHQ1
OXA58-1	GTTGGTATGTGGGTTTTGTTG
OXA58-2	CGTAGAGCAATATCATCACCAG
OXA58-probe	Cy5-TGCCACCACTTGCCCATCTGCC-BBQ-650
CTXM-1-1	AATCTGACGCTGGGTAAAGC
CTXM-1-2	GATATCGTTGGTGGTGCCATA
CTXM-1-probe	FAM-CCTGAATGCTCGCTGCACCGG-BHQ1
CTXM-9-1	CCGATCTGGTTAACTACAATCC
CTXM-9-2	GCTGGGCAATCAATTTGTTC
CTXM-9-probe	TexRed-CAACGGCACAATGACGCTGGC-BHQ2
CMY2-1	GATGCAGGAGCAGGCTATTC
CMY2-2	AACACGCCGTTAAACGTCTTAC
CMY2-probe	FAM-CCAATAACCACCCAGTCACGCAG-BHQ1
FOX-1	GTTCGAGATTGGCTCGGTCA
FOX-2	CACTGTAGGTGGCAAGCTCG
FOX-probe	HEX-TGGCTCACCTTGTCATCCAGC-BHQ1
ACT_MIR-1	ACTGGCAGCCGCAGTGGAAG
ACT_MIR-2	ACGTTAATCCASGTATGGTCCAGC
ACT_MIR-probe	FAM-AGACCCGCGTCGTYATGGCCTG-BHQ1
DHA-1	GGCGATATGCGTCTGTATGC
DHA-2	GTCAGCAACTGCTCATACGG
DHA-probe	TexRed-CCTGTTTGGTGCTCTGACCGC-BHQ2
mcr-1-1	ATGGCACGGTCTATGATACG
mcr-1-2	CACACCCAAACCAATGATACG
mcr-1-probe	FAM-ACCGACCAAGCCGAGACCAAGGA-BHQ1
mcr-2-1	GCCAACAGACACCATCTATC
mcr-2-2	TAGCCATTGAACTGCACATG
mcr-2-probe	HEX-ACCACCAAGCCGAGCGAGCG-BHQ1

a TexRed, Texas Red; BHQ1, black hole quencher 1; BHQ2, black hole quencher 2; FAM, 6-carboxyfluorescein; HEX, 6-carboxy-2,4,4,5,7,7-hexachlorofluorescein.

**TABLE 5:** Primers used for detection of class 1 integron-integrase gene (*intI1*) and the 16S rRNA gene

Primer	Sequence (5′–3′) (reference)
*intI1* F165	CGAACGAGTGGCGGAGGGTG (14)
*intI1* R476	TACCCGAGAGCTTGGCACCCA (14)
16S rRNA gene F919	GAATTGACGGGGGCCCGCACAAG (84)
16S rRNA gene R1378	CGGTGTGTACAAGGCCCGGGAACG (84)

### Isolation of antibiotic-resistant bacteria.

Samples were cultivated in tryptic soy broth (TSB; Merck, Darmstadt, Germany) containing low concentrations of imipenem, cefotaxime, or colistin to select resistant species. Low concentrations were used since antibiotics normally do not occur in lethal concentrations in the investigated environments, and in this way, the growth of susceptible strains should be inhibited, while strains with only decreased susceptibility and resistance may still be isolated (80). In this respect, 5 ml of TSB containing 2-μg ml^−1^ imipenem or 6-μg ml^−1^ cefotaxime was inoculated with 100 μl of prepared samples and 1 ml of TSB containing 10-μg ml^−1^ colistin was inoculated with 50 μl of prepared sample to allow, respectively, the isolation of carbapenemase- and ESBL-producing species as well as bacteria resistant to colistin. The inoculated broths were incubated for 24 h at 30°C in an orbital shaker at 300 rpm. In the case of growth, the broth cultures were streaked out on MacConkey agar (Carl Roth, Karlsruhe, Germany) with antibiotic disks (24 h at 30°C) of either imipenem, cefotaxime, or colistin, depending on the antibiotic used in the broth, and on chromID agar for the isolation of carbapenemase-producing (chromID CarbaSmart; bioMérieux, Nürtingen, Germany) and ESBL-producing (chromID ESBL; bioMérieux) bacteria (24 h at 37°C). After incubation, colonies in the area of the antibiotic disk were differentiated by morphology and subcultivated to obtain pure isolates, which were applied to the Vitek 2 system for the determination of species and antimicrobial susceptibility testing (see below).

### Culture-based determination of species and antimicrobial susceptibility testing.

After 24 h of incubation on MacConkey agar at 30°C, fresh isolates were applied to a Vitek 2 compact system (bioMérieux) for the determination of bacterial species and resistance to antibiotics, according to the manufacturer’s instructions. Clinical resistance was assessed on the basis of EUCAST breakpoints (81). The Vitek 2 GN, Vitek 2 AST N-214 (ESBL test, ampicillin, ampicillin/sulbactam, piperacillin-tazobactam, cefuroxime, cefuroxime axetil, cefpodoxime, cefotaxime, ceftazidime, ertapenem, imipenem, meropenem, gentamicin, ciprofloxacin, moxifloxacin, tetracycline, tigecycline, trimethoprim-sulfamethoxazole), and Vitek 2 AST N-248 (piperacillin, piperacillin-tazobactam, cefotaxime, ceftazidime, cefepime, aztreonam, imipenem, meropenem, amikacin, gentamicin, tobramycin, ciprofloxacin, moxifloxacin, tigecycline, fosfomycin, colistin, trimethoprim-sulfamethoxazole) systems were used for the identification and analysis of the antibiotic resistance of Gram-negative bacteria, without prior determination of Gram staining status. After determination of antibiotic resistances, DNA was extracted from the identified isolates by a standard heat treatment (82) and qPCR was performed as well. Bacterial strains confirmed to produce an ESBL by use of the Vitek 2 system were screened for *bla* genes belonging to the *bla*_CTX-M_, *bla*_CMY-2_, *bla*_FOX_, *bla*_ACT/MIR_, and *bla*_DHA_ families. Carbapenem-resistant isolates were screened for *bla*_VIM_, *bla*_NDM_, *bla*_KPC_, *bla*_GES_, *bla*_OXA-48_, *bla*_OXA-58_, and *bla*_OXA-23_, while colistin-resistant isolates were analyzed for the *mcr-1* and *mcr-2* genes.

### Reduction of antibiotic-resistant bacteria in washing machines and dishwashers.

For investigating the antimicrobial effect of both washing machines and dishwashers, an antibiotic-resistant strain and a susceptible strain of Escherichia coli (susceptible strain ATCC 10536 and a resistant strain isolated from a shower drain), Klebsiella pneumoniae (susceptible strain ATCC 13883 and an ESBL-producing strain isolated from a wastewater treatment plant), and Staphylococcus aureus (susceptible strain ATCC 6538 and methicillin-resistant Staphylococcus aureus [MRSA] strain CCUG35601) were used. Artificially contaminated cotton test swatches were prepared as described by Honisch et al. (41) for the analysis of the laundering process. In this respect, subcultures of each strain were prepared, the second subculture was centrifuged for 5 min at 4,696 × *g*, and the supernatant was discarded. After that, the resulting pellet was suspended in sterile 0.154 M sodium chloride, the suspension was centrifuged, and the pellet was suspended in 10 ml of fetal bovine serum and decanted again. Sterile cotton swatches of 1 cm^2^ complying with DIN standard 53919 (wfk 10 A 100% cotton; wfk Testgewebe GmbH, Brüggen, Germany) were added to the bacterial suspension for 10 min, followed by drying in open sterile petri dishes for 2 h at 37°C and storage at −18°C until use. In contrast, stainless steel biomonitors of DIN standard EN 10088-3 were artificially contaminated and used for the dishwasher tests, as described by Brands et al. (78). Instead of liquid cultures, surface cultures on TSA, followed by second and third subcultures, were prepared by streaking out bacterial colonies using a sterile inoculation loop. Triplicates of the third subculture were used for the contamination of biomonitors. The plates were rinsed with 10 ml of sterile 0.9% sodium chloride, followed by centrifugation for 5 min at 4,696 × *g*. The supernatant was discarded, and the pellet was suspended in 10 ml of soil matrix containing 0.6% bovine serum albumin (BSA; reference number A1391.0100; AppliChem GmbH, Darmstadt, Germany), 1% mucin (reference number 8494.1; Carl Roth GmbH+Co. KG, Karlsruhe, Germany), and 3% corn starch (reference number 9444.1; Carl Roth GmbH+Co. KG), used in DIN standard 10512 (Deutsches Institut für Normung e. V., 2008). Before inoculation, the biomonitors were cleaned in an ultrasound bath (reference number USC900TH; VWR, Darmstadt, Germany) and autoclaved (model Systec VX-65 autoclave; Systec GmbH, Linden, Germany). Afterwards, 100 μl inoculation solution was applied to the middle area of the biomonitors and spread evenly on the surface using a sterile inoculation loop (Sarstedt, Nümbrecht, Germany). The biomonitors were dried for 4 h at 22°C and 70% relative humidity in a climate chamber (reference number HPP110; Memmert GmbH+Co. KG, Schwabach, Germany) and stored in individual test tubes at 5°C until use. The dishwasher tests were performed in an automated dishwasher (model GSL-2; Miele & Cie. KG, Gütersloh, Germany) with six repetitions each, and the following conditions were tested: a 5-min main wash at 45°C with a rinsing cycle at 50°C, a 15-min main wash at 60°C with a rinsing cycle at 50°C, and a 90-min main wash at 45°C with a rinsing cycle at 50°C.

To determine the effect of domestic laundering on antibiotic-resistant strains, test runs were performed in a lab-scale washing machine (Rotawash) using the method of Schages et al. (44). The 1-liter vessels of the lab-scale washing machine represent the drum of a domestic washing machine, and the parameters ballast load of textiles, soil ballast, and detergent were adjusted to a volume of 0.5 liter of water, while eight steel beads simulated the mechanical action of a washing machine. The ballast load consisted of 96.5 g cotton (wfk 10 A; 170 g/m^2^; wfk Testgewebe GmbH) and 3.5 g SBL2004 (wfk Testgewebe GmbH) as organic soil ballast. All tests were performed in triplicate with six contaminated swatches included in each test run, and the 30- and 60-min main washes were tested at 30°C and 40°C using 2.7 g IEC-A* base powder (wfk Testgewebe GmbH) as an activated oxygen bleach (AOB)-free detergent. Besides the main wash, the effect of the rinsing cycle was investigated with and without the addition of quaternary ammonium compounds (QACs), using 0.02% benzalkonium chloride (BAC).

After the tests, the logarithmic reduction (LR) was determined as described by Block et al. (83): LR = *K*_0_ − *K_S_*, where *K*_0_ is the initial load on the swatches/biomonitors before laundering/automated dishwashing and *K_S_* is the remaining load on the swatches/biomonitors after laundering/automated dishwashing.

### Data analysis.

Statistical analyses were performed using GraphPad Prism software (GraphPad Software Inc.). Data were expressed as the means ± standard errors. Data were log transformed prior to statistical analyses to meet the assumptions of normality and the homogeneity of variance, when needed. The absolute and relative abundance of *bla* genes and *intI1* genes and the phenotypic resistance of bacteria in SD, WM, and DW samples were normally distributed after log transformation (Shapiro-Wilk test). Thus, statistically significant differences were assessed using two-way analysis of variance (ANOVA), and *post hoc* Tukey’s multiple-comparison test was performed to identify significant differences between the ARG groups in SD, WM, and DW samples (*P ≤ *0.05). Since phenotypic resistances were normally distributed as well, significant differences between resistances to the antibiotics included in the study were determined as mentioned above. Due to a lack of a Gaussian distribution, Spearman correlation analysis (*P ≤ *0.05) was performed to analyze the co-occurrence of ARGs and to determine the relation between phenotypic and genotypic resistance. Log-transformed data from the main wash and dishwasher experiments were normally distributed, and thus, a two-way ANOVA and *post hoc* Tukey’s multiple-comparison test were performed to identify significant differences, while the nonparametric Kruskal-Wallis test was performed to compare the logarithmic reductions in the rinsing cycle tests.

## Supplementary Material

Supplemental file 1
