# Supplementary material for: The Household Resistome: Frequency of β-Lactamases, Class 1 Integrons, and Antibiotic-Resistant Bacteria in the Domestic Environment and Their Reduction during Automated Dishwashing and Laundering
Source: Appl Environ Microbiol. 2020 Nov 10;86(23):e02062-20. doi: 10.1128/AEM.02062-20 (PMC7657631; doi:10.1128/AEM.02062-20)
Supplement: Supplemental file 1 [file AEM.02062-20-s0001.pdf]

Supplemental material

Resistant bacteria screened via qPCR

Table S1: Resistances and beta-lactamase genes determined in strains isolated using subinhibitory concentrations of imipenem or colistin from shower drain, washing machine and dishwasher samples (PIP/TAZ=piperacillin/tazobactam, CTX/CAZ=cefotaxime/ceftazidime, IPM/MEM=imipenem/meropenem, CIP=ciprofloxacin). The resistance phenotype was determined by VITEK 2 system (ESBL) or according to international recommendations (MDR). A * marks strains which were not classified as intermediate but had a decreased sensitivity (only applies to imipenem and meropenem).

species	sample	resistance phenotype	resistance genes	resistances
Pseudomonas aeruginosa	shower drain	MDR	OXA-58, OXA-23	PIP/TAZ, CTX/CAZ, IPM/MEM
Acinetobacter lwoffii	shower drain		OXA-58, OXA-23	PIP/TAZ, IPM*
Escherichia coli	shower drain	MDR, ESBL	OXA-48, CTX-M-9	PIP/TAZ, CTX/CAZ, IPM/MEM
Pseudomonas aeruginosa	shower drain		OXA-58, OXA-23, OXA-48	IPM/MEM
Brevundimonas diminuta	shower drain		OXA-58	PIP/TAZ, IPM
Serratia marcescens	shower drain		OXA-58	-
Aeromonas hydrophila	shower drain		OXA-58, OXA-23, FOX	IPM/MEM
Pseudomonas aeruginosa	shower drain		GES	CTX/CAZ, IPM/MEM
Enterobacter cloacae	shower drain		GES, ACT/MIR	IPM*
Morganella morganii	shower drain	ESBL	OXA-48, ACT/MIR, DHA	PIP/TAZ, CTX/CAZ
Delftia acidovorans	shower drain		GES	CT
Citrobacter braakii	shower drain	MDR	OXA-23	CTX/CAZ, IPM/MEM

Pseudomonas fluorescens	washing machine		OXA-58, VIM	PIP/TAZ, IPM*
Pseudomonas fluorescens	washing machine		OXA-58, OXA-23, GES	IPM
Pseudomonas putida	washing machine		OXA-23	IPM/MEM*
Pseudomonas fluorescens	washing machine		OXA-58	IPM/MEM*
Pseudomonas aeruginosa	washing machine		OXA-58, OXA-48	IPM/MEM
Pseudomonas aeruginosa	washing machine		OXA-58	IPM/MEM
Pseudomonas putida	dishwasher		GES	IPM*/MEM*
Pseudomonas fluorescens	dishwasher		OXA-58, VIM	MEM*
Pseudomonas aeruginosa	dishwasher		OXA-23	IPM/MEM*
Pseudomonas mendocina	dishwasher		OXA-48	IPM/MEM*
Citrobacter freundii	dishwasher		OXA-58, OXA-23	PIP/TAZ, CTX/CAZ

Table S2: Resistances and beta-lactamase genes determined in strains isolated using subinhibitory concentrations of cefotaxime from shower drain, washing machine and dishwasher samples (PIP/TAZ=piperacillin/tazobactam, CTX/CAZ=cefotaxime/ceftazidime, IPM/MEM=imipenem/meropenem, ETP=ertapenem, CIP=ciprofloxacin, CXM=cefuroxime). The resistance phenotype was determined by VITEK 2 system (ESBL) or according to international recommendations (MDR). A * marks strains which were not classified as intermediate but had a decreased sensitivity (only applies to imipenem and meropenem).

**species**	**sample**	**resistance phenotype**	**resistance genes**	**resistances**
Citrobacter freundii	shower drain	MDR; ESBL	CTX-M-1, CMY-2	PIP/TAZ, CTX/CAZ
Pseudomonas putida	shower drain		GES	MEM
Serratia ficaria	shower drain	MDR	CMY-2, GES	CTX/CAZ, MEM
Enterobacter cloacae	shower drain	MDR	ACT/MIR	PIP/TAZ, CTX/CAZ, ETP

Citrobacter freundii	shower drain	ESBL, MDR	CMY-2	PIP/TAZ, CTX/CAZ
Sphingomonas paucimobilis	shower drain		GES	MEM, CIP
Escherichia coli	shower drain	ESBL, MDR	CTX-M-1, GES	PIP/TAZ, CTX/CAZ, CIP
Enterobacter cloacae	shower drain	MDR	ACT/MIR	PIP/TAZ, CTX/CAZ
Pseudomonas putida	shower drain		GES	MEM
Citrobacter freundii	washing machine	ESBL	CMY-2	PIP/TAZ, CTX/CAZ
Achromobacter denitrificans	washing machine		GES	PIP/TAZ, IPM, CIP
Achromobacter denitrificans	washing machine		GES	PIP/TAZ, IPM/MEM, CIP
Pseudomonas putida	washing machine		CTX-M-1, OXA-48, GES	MEM
Enterobacter cloacae complex	washing machine		CTX-M-1, ACT/MIR	
Acinetobacter baumannii	dishwasher		ACT/MIR	CTX/CAZ
Enterobacter cloacae	dishwasher		ACT/MIR	PIP/TAZ, CTX/CAZ
Enterobacter cloacae	dishwasher		ACT/MIR, GES	PIP/TAZ, CTX/CAZ, ETP
Enterobacter cloacae	dishwasher	ESBL	CTX-M-1, ACT/MIR	PIP/TAZ, CTX/CAZ

Effect of laundering and automated dishwashing on susceptible strains

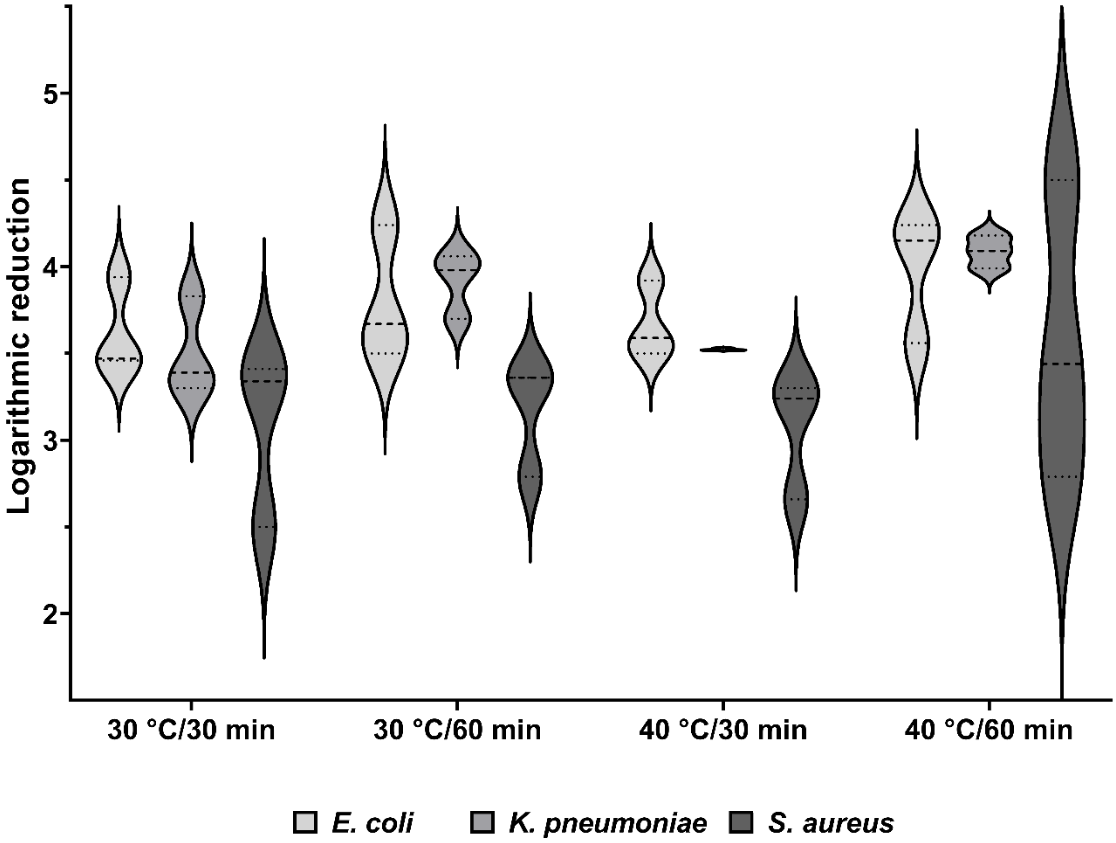

Figure S1: Impact of the main wash of the laundering process simulated using the Rotawash on susceptible strains of *Escherichia coli*, *Klebsiella pneumoniae* and *Staphylococcus aureus* with AOB-free detergent.

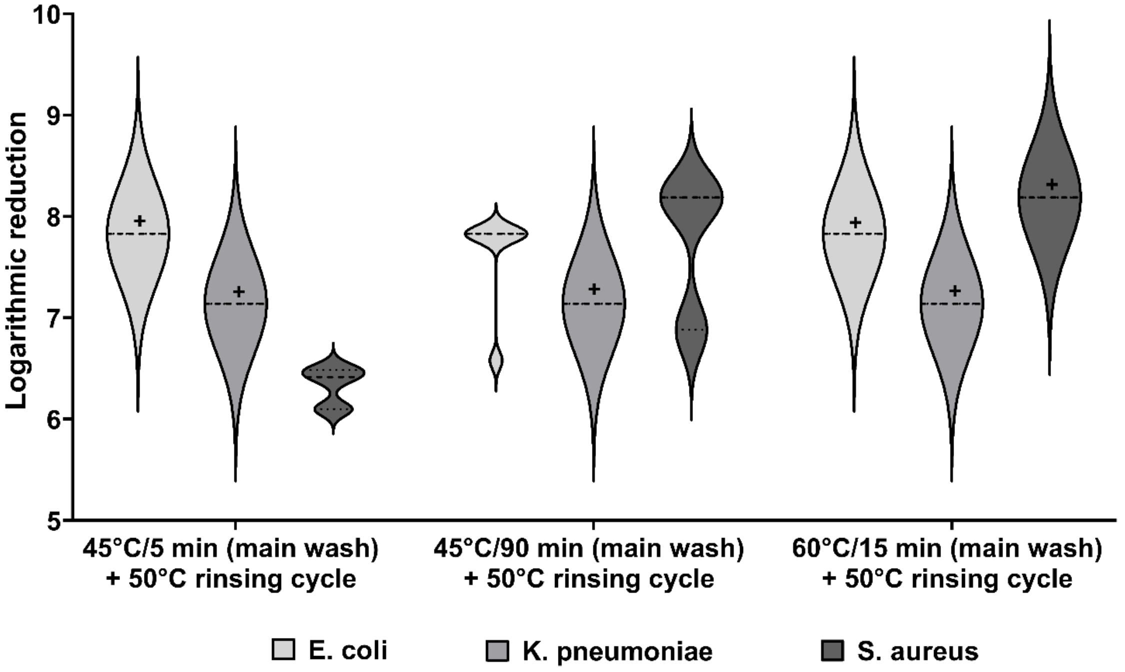

Figure S2: Impact of automated dishwashing on susceptible strains of *Escherichia coli*, *Klebsiella pneumoniae* and *Staphylococcus aureus* with detergent. The different values for LR max. [+] (indicating a complete reduction of the microbial load) were obtained due to different initial loads on the biomonitors.
